# Assessing Interest in a Peer Support Person for Patients Experiencing Early Pregnancy Loss: Results from a National Survey

**DOI:** 10.1089/whr.2023.0132

**Published:** 2024-03-27

**Authors:** Carmen Conroy, Tanya Jain, Tuo Lin, Sheila K. Mody

**Affiliations:** ^1^ School of Medicine, University of California San Diego, San Diego, California, USA.; ^2^ Department of Biostatistics, University of California San Diego, San Diego, California, USA.; ^3^ Division of Complex Family Planning, Department of Obstetrics, Gynecology and Reproductive Sciences, University of California San Diego, San Diego, California, USA.

**Keywords:** early pregnancy loss, miscarriage, self-compassion, resilience

## Abstract

**Introduction:**

The use of a peer support person as an intervention for early pregnancy loss (EPL) is not well studied. In addition, limited literature exists regarding the type of support patients need when experiencing EPL. The objective of this study is to quantify interest in a peer EPL support person intervention, to assess the types of support desired following EPL, and to investigate if there is an association between self-compassion or resilience and coping ability post-EPL.

**Methods:**

We conducted a cross-sectional, web-based survey with 110 individuals who experienced EPL in the past 2 years. Questions explored interest in a peer EPL support person and different types of support, as well as perceived self-compassion and resilience. Analyses of variance were used to test if interest in the peer support intervention and in different types of support varied by demographics, while linear regression modeling was used to test the relationship between self-compassion, resilience, and coping ability.

**Results:**

Nearly all participants (98.2%, *n* = 108) were interested in peer support. The majority (31.8%, *n* = 35) of participants prioritized informational and educational support at the time of their EPL and in the months following. There was a positive relationship between self-compassion scores and ability to cope with EPL (*p* = 0.2) and between resilience scores and coping ability (*p* < 0.05).

**Conclusions:**

Almost all participants were interested in a peer support person for coping with EPL. Given the types of support participants identified in this study, a peer support person may provide emotional and informational support as well as resilience training.

## Introduction

An estimated 6.5 million early pregnancy losses (EPLs) occur every year in the United States, and as many as one quarter of women report ever having experienced an EPL.^1^ Despite the prevalence of EPL, limited data exist regarding the specific needs of individuals following EPL. Recent research has begun the work of characterizing the emotional, physical, and even financial burden of miscarriage.^2–4^ In addition, previous studies have begun to illuminate patient emotional needs following EPL, demonstrating that while there is often a lack of social literacy surrounding miscarriage,^5,6^ interpersonal relationships can offer indispensable support following EPL.^7,8^ These interpersonal networks may serve to buffer the support provided by existing pregnant loss support organizations.

This desire for increased access to on-call, patient-centered social, emotional, and informational support could possibly include meeting with a peer support person or engaging in self-compassion and resilience training as an EPL coping strategy.^1,9,10^ However, there is a need for a larger scale study to quantify interest in such a peer support person intervention, in different types of support at the time of and following EPL, and in the practice of self-compassion or resilience as an EPL coping strategy.

In this study, we measured interest in a peer support person intervention and characterized the relative demand for different types of EPL support during and following EPL. We also sought to determine if various types of support needs changed across different demographic groups experiencing EPL. In addition, we sought to determine whether certain factors such as perceived self-compassion or resilience may be correlated with improved ability to cope with EPL. These data will inform the potential role that a peer support person could fulfill as an intervention for patients experiencing EPLs in the future.

This national survey allows for better determination of widespread interest in a peer support program and of programmatic input (PI) for such a program, including a possible self-compassion or resilience component. The findings of this study will allow us to draw preliminary conclusions about the support needs and desires of different demographic groups, which can allow us to better tailor future peer support person interventions.

## Methods

### Study design

This study was a cross-sectional, web-based survey of 110 EPL patients in the United States. Participants confirmed that they met the eligibility criteria by participating in a brief prescreening survey in the online survey form. All participants provided informed consent.

#### Participants did not receive formal peer support at the time of the survey

Survey questions explored interest in a peer EPL support person, input for the creation of such a support intervention, and perceived self-compassion and ability to cope with adversity. The survey also contained demographic questions about relevant topics such as participants' insurance status, partnered status, and self-identified race and ethnicity. The self-compassion and resilience survey questions were based on previously validated surveys, known as the State Self-Compassion Scale (SSCS)^11^ and the Brief Resilient Coping Scale (BRCS),^12^ respectively. The study was approved by our institution's Human Research Protection Program.

### Sampling and eligibility criteria

We recruited English-speaking individuals between 18 and 50 years of age who experienced EPL within the 2 years before study enrollment. Participants were recruited from EPL-related social media groups and from an EPL clinic at an academic medical center in La Jolla, CA.

### Data collection

Participants completed a web-based, 44-item survey. The survey was hosted on Research Electronic Data Capture, a secure, web-based application designed to support data capture for research studies.^13^ The survey consisted of six sequential instruments (Supplementary Appendix SA1): (1) a 4-item Peer Support Interest Assessment (PSIA), which queried general interest in access to a peer support person; (2) a 7-item PI instrument, which asked participants to rate the most useful type of support for navigating their EPL; (3) a 12-item SSCS, a validated tool for measuring self-compassion; (4) a 4-item BRCS, a validated tool for measuring emotional resilience; (5) a 2-item Early Pregnancy Loss Coping Scale (EPLCS) questionnaire, which aimed to assess self-reported healing post-EPL; and (6) a 14-item demographic questionnaire, which asked participants to self-identify different demographic factors, including race, ethnicity, income, insurance status, and educational status.

Instruments 1, 3, 4, and 5 used a 5-point Likert scale ranging from 1 (“strongly disagree,” “almost never,” or “does not describe me at all”) to 5 (“strongly agree,” “almost always,” or “describes me very well”). Instruments 2 and 6 allowed participants to select one option from a prewritten dropdown menu. A write-in option was provided after each instrument and at the end of the survey to allow participants to clarify any responses or provide supplement information if they desired. Participants who completed the survey were compensated for their time with a $10 electronic gift card.

### Analysis

The survey data were analyzed using Stata 18 software.^14^ In addition to descriptive statistics, one-way analyses of variance (ANOVAs) were used to test if there were differences in rated interest in the peer support intervention between different demographic groups, and Pearson chi-squared tests were used to assess differences in the type of support most requested by different demographic groups. In addition, we used linear regression modeling to test the relationship between self-compassion (SSCS scores) and coping (EPLCS scores), as well as to test the relationship between resilience (BRCS scores) and coping.

## Results

The first four survey questions formed the PSIA, a questionnaire that aimed to gauge interest in a peer support intervention by asking participants to score different statements regarding a peer support intervention on a Likert scale from 1 (strongly disagree) to 5 (strongly agree). The average score in response to all four items in the PSIA was tabulated and averaged to obtain an overall average PSIA score for all participants (mean 4.18, standard deviation 0.89), which fell in the “strongly agree” range (4–5). This high PSIA average across participants indicates that, on average, all participants strongly agree that they would have wanted a peer support intervention had it been available, that they believe a peer who has experienced EPL is uniquely poised to offer EPL support, and that they would recommend the use of a peer support person to others (Table 1).

**Table 1.** Rated Interest in Peer Support Based on a 4-Item Peer Support Interest Assessment

	Item 1: “I would have been interested in having access to a peer support person during my pregnancy loss.”	Item 2: “Someone who has also experienced a pregnancy loss understands more than other people in my life what it is like to have a pregnancy loss.”	Item 3: “I believe that a peer who has also experienced pregnancy loss could offer me special support during a pregnancy loss.”	Item 4: “I would be more likely to recommend the use of a peer support person as a pregnancy loss intervention if they had training in self-compassion practices than if they did not.”	Mean PSIA (SD PSIA)
Mean (SD)	4.20 (0.91)	4.26 (0.73)	4.11 (0.94)	4.17 (0.97)	4.18 (0.89)

Items were scored on a scale from 1 (strongly disagree) to 5 (strongly agree).

PSIA, Peer Support Interest Assessment; SD, standard deviation.

Furthermore, when disaggregated, participants across almost all distinct demographic groups—including race, ethnicity, age at the time of most recent EPL, annual income, highest education level attained, insurance coverage status, type of EPL management, location of EPL care, history of multiple EPLs, intension of pregnancy resulting in EPL, history of psychiatric diagnosis before EPL, and history of psychiatric diagnosis following EPL—reported high average PSIA scores in the “strongly agree” (4–5) range (Table 2). Importantly, American Indian/Alaska Native, Asian/Pacific Islander, and doctorate degree groups have *n* < 5 participants, which limits the statistical power of results for these groups and reinforces the need for additional research to center these groups and adequately represent their perspective.

**Table 2.** Demographic Subgroups and Their Corresponding Peer Support Interest Assessment Scores

Demographic group (***n*** = 110)	Mean PSIA score (SD)
Race	***p-* < 0.0001**
White (*n* = 38, 34.5%)	4.35 (0.65)
Black or African American (*n* = 67, 60.91%)	4.15 (0.50)
Asian/Pacific Islander (*n* = 1, 0.91%)	1.89 (1.69)^a^
American Indian/Alaska Native (*n* = 4, 3.64%)	3.83 (1.19)^a^
Ethnicity	***p* = 0.25**
Hispanic or Latino (*n* = 9, 8.18%)	4.35 (0.49)
Not Hispanic or Latino (*n* = 101, 91.8%)	4.17 (0.60)
Age at time of EPL	***p* = 0.65**
21–24 (*n* = 26, 23.6%)	4.15 (0.56)
25–27 (*n* = 35, 31.8%)	4.15 (0.59)
28–30 (*n* = 22, 20.0%)	4.32 (0.49)
30+ (*n* = 27, 24.5%)	4.14 (0.72)
Annual income	***p* = 0.14**
$40,000 (*n* = 8, 7.27%)	4.52 (0.49)
$40,000–90,000 (*n* = 18, 16.4%)	4.34 (0.66)
$90,000–170,000 (*n* = 75, 68.2%)	4.12 (0.60)
$215,000 (*n* = 9, 8.18%)	4.12 (0.50)
Highest education level attained	***p* = 0.20**
High school diploma or GED (*n* = 1, 0.91%)	5 (0)
Some college (*n* = 15, 13.6%)	4.29 (0.41)
College degree (*n* = 72, 65.5%)	4.15 (0.57)
Master's degree (18, 16.4%)	4.27 (0.74)
Doctorate (*n* = 4, 3.63%)	3.73 (1.19)^a^
Insurance coverage status	***p* = 0.006**
Public (*n* = 72, 65.5%)	4.14 (0.53)
Private/commercial (*n* = 29, 26.4%)	4.09 (0.89)
Military (TriCare) (*n* = 7, 6.36%)	4.75 (0.22)
Uninsured (*n* = 2, 1.81%)	4.92 (0.06)
EPL management	***p* = 0.62**
Expectant management (*n* = 35, 31.8%)	4.27 (0.49)
Medication management (*n* = 57, 51.8%)	4.11 (0.61)
D&C (*n* = 16, 14.5%)	4.23 (0.77)
Other (*n* = 2, 1.81%)	4.2 (0.66)
Location of EPL care	***p* = 0.61**
Emergency room (*n* = 14, 12.7%)	4.34 (0.45)
Community health center (*n* = 48, 43.6%)	4.18 (0.60)
Academic institution (*n* = 26, 23.6%)	4.03 (0.59)
Planned parenthood (*n* = 7, 6.36%)	4.20 (0.73)
Private practice (*n* = 9, 8.18%)	4.28 (1.00)
Other (*n* = 6, 5.45%)	4.31 (0.64)
History of multiple EPLs	***p* = 0.26**
Yes (*n* = 46, 41.8%)	4.13 (0.60)
No (*n* = 64, 58.2%)	4.25 (0.59)
Pregnancy that resulted in EPL	***p* = 0.05**
Intended (*n* = 66, 60%)	4.08 (0.62)
Unintended (*n* = 43, 39.1%)	4.32 (0.57)
Other (*n* = 1, 0.91%)	4.84 (0.13)
Psychiatric diagnosis before EPL	***p* = 0.35**
Yes (*n* = 78, 70.9%)	4.15 (0.57)
No (*n* = 32, 29.1%)	4.26 (0.65)
New psychiatric diagnosis since EPL	***p* = 0.25**
Yes (*n* = 60, 54.5%)	4.24 (0.45)
No (*n* = 50, 45.5%)	4.11 (0.76)

^a^ Denotes those demographic groups that do not fall within the “strongly agree” (4–5) range.

D&C, dilation and curettage; EPL, early pregnancy loss; GED, Graduate Education Diploma.

Upon analyzing the demographic groups using ANOVAs, there are no statistical differences between the subgroups within each demographic group with three exceptions: race, insurance coverage status, and intention of pregnancy that resulted in EPL (Table 2). In other words, those identifying with different races, participants with different insurers, and those who had intended versus unintended pregnancies varied significantly in their PSIA score, representing variance in how strongly they expressed interest in a peer support intervention. Interestingly, those who had unintended pregnancies reported slightly higher mean PSIA scores compared with their intended pregnancy counterparts. Perhaps unsurprisingly, those who reported that they were uninsured or had military-affiliated insurance reported slightly higher PSIA scores than their commercially insured counterparts. However, individuals reporting any insurance status or any intention behind the pregnancy that resulted in EPL still reported average PSIA scores in the “strongly agree” (4–5) range.

To explore which type of support was most sought after both at the time of experiencing EPL and in the months following, participants were asked to rank which type of support they viewed as most important during these two periods: informational/educational, physical, spiritual, support for their partner or family, or connection to outside resources. A write-in “other” option was also provided. The most highly requested type of support both at the time of EPL (*n* = 35, 31.8%) and in the months following (*n* = 35, 31.8%) was informational or educational support, which was defined as access to information regarding what to expect during EPL and recovery. In addition, the second-most highly requested type of support at both time points (*n* = 32, 29.1% at the time of EPL and *n* = 35, 31.8% in the months following) was emotional support.

Therefore, informational and emotional support was consistently rated as the most important type of support and remained stable in their relative demand over time.

**FIG. 1.**
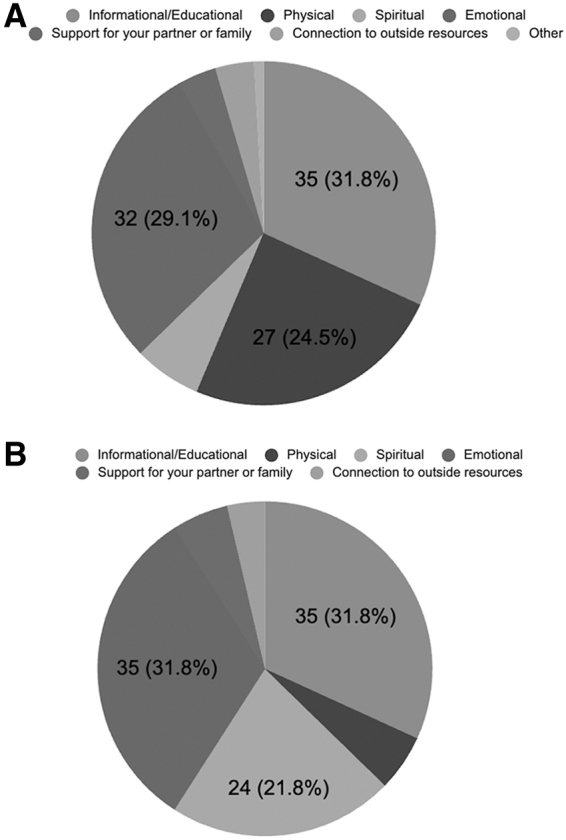
Relative percentages of different types of support ranked as most important **(A)** at the time of EPL and **(B)** in the months following EPL. Labels shown/110 (%) for the top three most requested types of support. EPL, early pregnancy loss.

Other types of support rated as most important fluctuated more between the time of EPL and the months following. For example, physical support was moderately rated (*n* = 27, 24.5%) as the most important type of support at the time of EPL, but less likely to be rated as most important in the months following EPL (*n* = 6, 5.5%). In contrast, few individuals (*n* = 7, 6.4%) rated spiritual support as most important at the time of EPL, but many individuals (*n* = 24, 21.8%) rated it as important in the months following. These results are summarized in Figure 1.

To explore if the type of support ranked as most important at the time of EPL varied according to demographic group, we used Pearson's chi-squared test to compare types of support ranked as most important between black or African American and white participants. Please note that other racial groups had *n* < 5 participants and therefore insufficient statistical power for comparison. Differences between black or African American and white participants did not differ significantly (*p* = 0.29). We also used Pearson's chi-squared test to compare types of support ranked as most important by those with private versus public insurance. The type of support ranked as most important at the time of EPL did not vary significantly (*p* = 0.77) between these groups either.

Lastly, we used Pearson's chi-squared test to compare types of support ranked as most important at the time of EPL by those belonging to different income brackets, ranging from no income to >$215,000 annually. We found that among individuals belonging to different income brackets, the type of support ranked as most important at the time of EPL did vary significantly (*p* = 0.002), with the lowest income groups being more likely to rank emotional support as more important than informational or educational support.

To assess the role that resilience and self-compassion might play in recovering from EPL, participants completed the validated BRCS and SSCS, respectively, in the online survey. As previously outlined in the literature, BRCS sum scores range from 4 to 20, with scores of 4–13 indicating low resilient coping, 14–16 indicating medium resilient coping, and 17–20 indicating high resilient coping.^12^ For the SSCS, scores from 1 to 2.49 are considered low, 2.5–3.5 considered moderate, and 3.51–5.0 considered high.^11^ The BRCS and SSCS scores were then compared against the EPL Coping Scale, an averaged score from a two-item questionnaire, which asked participants to rate on a 5-point Likert scale from 1 (strongly disagree) to 5 (strongly agree) the extent to which they feel they have processed and emotionally healed from their EPL.

**FIG. 2.**
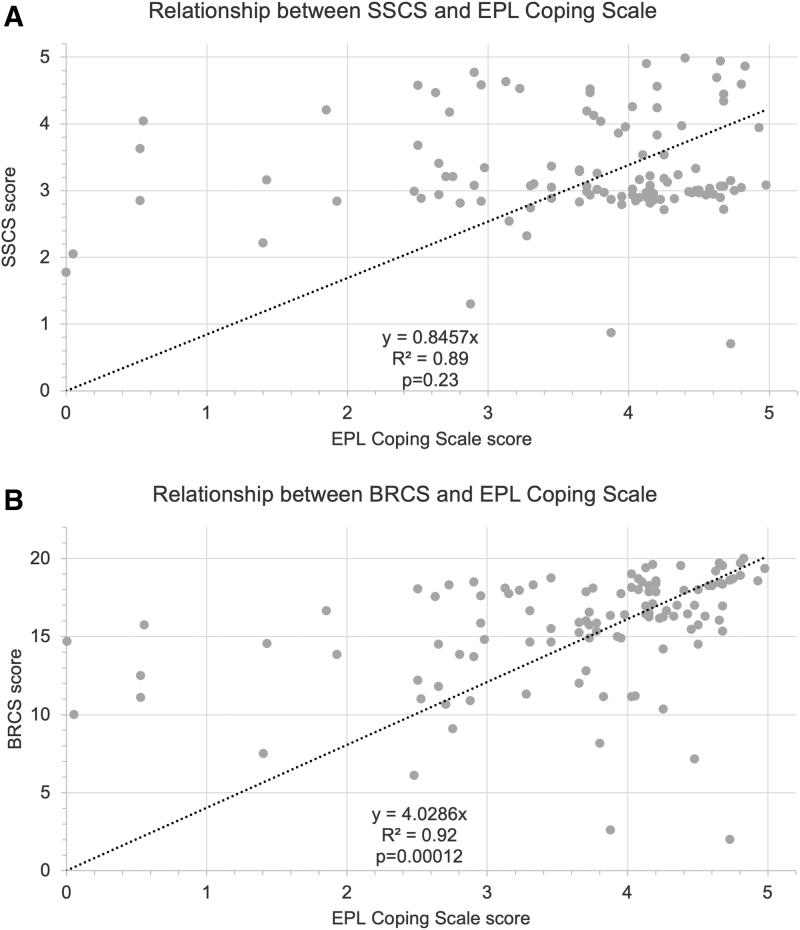
Relationship between **(A)** SSCS scores and EPLCS scores, as well as between **(B)** BRCS and EPLCS scores. BRCS, Brief Resilient Coping Scale; EPLCS, Early Pregnancy Loss Coping Scale; SSCS, State Self-Compassion Scale.

For both SSCS scores (*R^2^* = 0.89, *p* = 0.23) and BRCS (*R^2^* = 0.92, *p* = 0.00012) scores, there was a strong positive correlation with EPLCS scores. This indicates that people with higher self-compassion and higher resilience scores were more likely to rate themselves as having healed from their miscarriage. However, the positive relationship with EPLCS was only significant for BRCS scores (Fig. 2).

Of note, although all survey items provided opportunities for write-in responses, we did not receive any written responses from survey participants.

## Discussion

EPL is common, but there are few evidence-based, nonclinical support interventions. Several U.S.-based and international studies have highlighted the importance of interpersonal connection and communication in coping with pregnancy and infant loss, for the benefit of both the individuals experiencing the loss and their partner.^5,15–19^ Previous studies have shown that patients benefit from the aid of a doula—a nonclinical, emotional, and informational support person in the context of birth and abortion.^20,21^ However, few studies have explored the utility of this intervention in the context of EPL or researched the use of support persons who more specifically have experienced EPL themselves. In addition, although self-compassion and resilience are important outcomes in many interventions aimed at improving well-being, they have not been studied within the context of EPL.^22–24^

As a first step in exploring this idea, we sought to query interest in an EPL peer support person. Our data show that there is near universal interest in such an intervention, underscoring the current dearth of available resources for coping with EPL. A previous qualitative investigation has shown that individuals overwhelmingly feel that they would benefit from therapy or support groups, but find these support modalities to be financially, geographically, or otherwise inaccessible.^1^ In providing emotional, informational, and physical support, the use of a peer support person might help offset some of these demands. In terms of where peer support individuals might be recruited from, our study shows that the majority of individuals who experienced EPL are interested (82.7%, *n* = 91) in serving as peer support people themselves.

Further corroborating past qualitative findings,^1^ our studies show that the types of support most often ranked as most important are informational or educational support and emotional support, which could feasibly be provided by a trained peer support person. Since BRCS scores were significant correlated with the ability to cope post-EPL, a resilience component to any peer support person training could be helpful to future beneficiaries of such a program.

One limitation of this study was the reductive nature of asking individuals to rank their level of emotional healing numerically as in the EPLCS. This very narrowly captures individuals' emotions as a singular point in time; however, we attempted to attenuate this shortcoming by allowing participants to write in more detailed explanations below each EPLCS item. In addition, our study may be biased as our study sample was largely derived from social media. Another limitation to this study is the relatively small sample size, which limited full representation of certain demographic groups, and was constrained to those living in the United States, thus limiting generalizability of the findings. This may reduce the generalizability of the findings to groups who were represented in especially small numbers, such as those with Asian, Pacific Islander, or Native American ancestry.

To characterize the needs of these groups, and other minority populations with limited representation, they should be centered in future research and disaggregated to assess for intergroup variability. In addition, there may be unique support needs of certain groups—such as those who conceive *via in vitro* fertilization, those who experience multiple miscarriages, queer or same-sex couples, or single parents, for example—whose unique needs are not captured by this study. Further studies will explore specific support needs of these subgroups in higher granularity.

A strength of this study includes the assessment of self-compassion and resilience as correlates of coping post-EPL, as these skills are well-studied as markers of emotional well-being. The development of stronger self-compassion and resilience is an important outcome that should potentially be assessed in the future before and after participation in a possible peer support person program, as they may provide a measurement of well-being and coping post-EPL.

Overall, our findings will inform the curricular content of a future peer support intervention training module, which may be developed to include self-compassion and perhaps more importantly resilience training.

## Conclusions

There is high interest across almost all demographic groups in a peer support person intervention for use during and after EPL. This type of support person could help with the demand for informational and emotional support at the time of EPL, as well as spiritual support in the months following EPL. Access to a peer support person could furthermore provide an alternative to other financially or geographically inaccessible support tools following EPL. In addition, this peer support person could help individuals develop resilience and self-compassion to aid in coping with their EPL.

## Supplementary Material

Supplemental data

## Supplementary Material

Supplementary Appendix SA1

## Abbreviations Used

ANOVAs: analyses of variance
BRCS: Brief Resilient Coping Scale
D&C: dilation and curettage
EPL: early pregnancy loss
EPLCS: Early Pregnancy Loss Coping Scale
PI: programmatic input
PSIA: Peer Support Interest Assessment
SD: standard deviation
SSCS: State Self-Compassion Scale
