# Supplementary material for: Assessing Interest in a Peer Support Person for Patients Experiencing Early Pregnancy Loss: Results from a National Survey
Source: Womens Health Rep (New Rochelle). 2024 Mar 27;5(1):268–75. doi: 10.1089/whr.2023.0132 (PMC10979676; doi:10.1089/whr.2023.0132)
Supplement: Supplemental data [file Supp_AppS1.pdf]

Appendix 1: Online Survey

Peer Support Interest Assessment
For the following questions, a peer support person can be defined as another individual who has experienced a pregnancy loss) and received basic training on how to support people currently experiencing or pregnancy loss.

Please score the items below on a scale from 1 (strongly disagree) to 5 (strongly agree).

1. I would have been interested in having access to a peer support person during my pregnancy loss.

2. Someone who has also experienced a pregnancy loss understands more than other people in my life what it is like to have a pregnancy loss.

3. I believe that a peer who has also experienced pregnancy loss could offer me special support during a pregnancy loss.

4. I would be more likely to recommend the use of a peer support person as a pregnancy loss intervention if they had training in self-compassion practices than if they did not.

Programmatic Input
For the following questions, a peer support person can be defined as another individual who has experienced a pregnancy loss and received basic training on how to support people currently experiencing.

5. Please select the main kind of support you would want to receive from a peer support person **at the time of your pregnancy loss**.

- Emotional (someone to talk to about the emotional aspects)
- Physical (a hand to hold or help with breathing techniques during the procedure, etc.)
- Informational/educational (information on what to expect and recovery)
- Spiritual (guidance on meditation practices, including spirituality in your healing, etc.)
- Support for your partner or family
- Connection to outside resources (support groups or social media resources)
- Other (write in)

6. Please select the main kind of support you would want to receive from a peer support person **in the months following your pregnancy loss**.

- Emotional (someone to talk to about the emotional aspects)
- Physical (a hand to hold or help with breathing techniques during the procedure, etc.)
- Informational/educational (information on what to expect and recovery)
- Spiritual (guidance on meditation practices, including spirituality in your healing, etc.)
- Support for your partner or family
- Connection to outside resources (support groups or social media resources)

- Other (write in)

7. Please select what would be the most important aspect of training for a peer support person.
- Training on confidentiality and privacy
- Basic medical education on miscarriage
- Training on how to provide physical support, pain management, use of essential oils, and breathing techniques
- Other (write in)

8. Which format would you prefer to receive support from a peer support person **at the time of your pregnancy loss**?
- Texting
- Phone call
- Video conference call (Zoom)
- Email
- In person
- Other (write in)

9. Which format would you prefer to receive support from a peer support person **in the months following your pregnancy loss**?
- Texting
- Phone call
- Video conference call (Zoom)
- Email
- In person
- Other (write in)

10. How would you want to be connected with a peer support person?
- Sign up on website
- Through a social worker
- Through a doctor's office
- Other

11. Would you consider being a support person for someone else?
- Yes
- No
- Maybe

List or explain any other thoughts you have related to the possible implementation of a peer support program for pregnancy loss. This can include your preferences and suggestions for training and vetting of volunteers, concerns you have about the program, or things you think are important to emphasize or keep in mind as we create the program. (write in)

State Self-Compassion Scale (SSCS)
Please rate each item below from 1 (almost never) to 5 (almost always). Respond as you would have at the time of your pregnancy loss.

12. When I fail at something important to me, I become consumed by feelings of inadequacy.
13. I try to be understanding and patient towards those aspects of my personality I don't like.

14. When something painful happens, I try to take a balanced view of the situation.
15. When I'm feeling down, I tend to feel like most other people are probably happier than I am.
16. I try to see my failings as part of the human condition.
17. When I'm going through a very hard time, I give myself the caring and tenderness I need.
18. When something upsets me, I try to keep my emotions in balance.
19. When I fail at something that's important to me, I tend to feel alone in my failure.
20. When I'm feeling down, I tend to obsess and fixate on everything that's wrong.
21. When I feel inadequate in some way, I try to remind myself that feelings of inadequacy are shared by most people.
22. I'm disapproving and judgmental about my own flaws and inadequacies.
23. I'm intolerant and impatient towards those aspects of my personality I don't like.

Brief Resilient Coping Scale (BRCS)

Please rate each below from 1 (does not describe me at all) to 5 (describes me very well).

24. I look for creative ways to alter difficult situations.
25. Regardless of what happens to me, I believe I can control my reaction to it.
26. I believe I can grow in positive ways by dealing with difficult situations.
27. I actively look for ways to replace the losses I encounter in life.

EPL Coping Scale (EPLCS)

Please rate each below from 1 (does not describe me at all) to 5 (describes me very well).

28. I feel that I have processed my most recent pregnancy loss.
29. I feel that I have emotionally healed from my most recent pregnancy loss.

Demographic Information and Past Medical/Obstetric History

Please answer questions the following with respect to your most recent pregnancy loss if you have had more than one pregnancy loss.

30. What was your age at the time of your most recent pregnancy loss?
(drop down with ages 18-50)
31. Select your race
    a. American Indian/Alaska Native
    b. Asian
    c. Native Hawaiian or Other Pacific Islander
    d. Black or African American
    e. White
    f. Other (write)
32. Select your ethnicity
    a. Hispanic or Latino
    b. Not Hispanic or Latino
33. Please select the gender with which you most identify.
    a. Female
    b. Male
    c. Nonbinary
    d. Other (write in)
34. Which is your state of residence? (drop down)
35. What is your approximate annual income after taxes?
    a. 0-$10,000
    b. $10,000-40,000

    c. $40,000-90,000
    d. $90,000-170,000
    e. $170,000-215,000
    f. >$215,000

36. What is your highest level of education?
    a. Some high school
    b. High school diploma or GED
    c. Some college
    d. College degree
    e. Masters degree
    f. Doctorate
37. Select your primary insurance coverage.
    a. Private (United, Aetna, Blue Cross, etc.)
    b. Public (Medicaid)
    c. Military (TriCare)
    d. Uninsured
38. How was your pregnancy loss managed?
    a. Pregnancy spontaneously passed
    b. Medication management
    c. Dilation and curettage
    d. Other (write in)
39. In what type of setting did you receive medical care for your pregnancy loss?
    a. Emergency room
    b. Community health center
    c. Academic institution
    d. Planned Parenthood
    e. Private practice
    f. Other (write in)
40. Have you had more than one pregnancy loss?
    a. Yes
    b. No
41. Do you have any children?
    a. Yes – born prior to my most recent pregnancy loss
    b. Yes – born after my most recent pregnancy loss
    c. No
    d. Other: write in
42. Was your pregnancy that result in pregnancy loss intended or unintended?
    a. Intended
    b. Unintended
    c. Other: write in
43. Did you have any mental health condition(s) prior to your pregnancy loss?
    a. Yes – depression
    b. Yes – anxiety
    c. Other (write in)
    d. No
44. Since your pregnancy loss, have you been diagnosed with new mental health condition(s)?
    a. Yes – depression
    b. Yes – anxiety
    c. Other (write in)
    d. No
